# Medical Education

**Published:** 1866-09

**Authors:** 


### Editorial Department.

### Medical Education.

Medical Education is the subject everywhere selected on which to base a discourse upon lecture terms in colleges, advantage of public over private instruction, capacity of teachers, preliminary preparation, length of term of study, board of censors for granting diplomas, etc., etc. It is proposed to omit all these topics for the present, and show that a different use can be made of the same text. It is popularly regarded as *education* for a young man to pursue the usual collegiate course and graduate, and that no other plan than this can educate him. To read the old writers among the Greeks, and Cicero, Virgil, Sallust and Cæsar among the Latins, is certainly regarded as essential to education. In the same way medical education seems to be looked upon as dependent upon the lecture course, the eminence of the Professors and the standing and attainment of the private preceptor, and to consist in what a young man learns the few years before he receives his diploma. We hope to correct this view in own minds so far as it is entertained from long habit of thought and feeling, and show how we arrive at the conclusion that education is not the growth of a few years' juvenile pupilage at a literary or medical college, but rather the product of a life of study and observation.

Physicians are constantly writing and talking about the deficiencies in our plans of medical education, of the low standard of attainment and of the easy admission of young men to the toils and honors of our profession. Has it ever occurred to them that the young man of smallest attainment and the young man of the greatest, are separated from each other only by a difference so small that a few months of study can change the scale? Have they ever observed the boy who graduated with the highest college honors a few years later in life, outdone and excelled by the one who had no class rank at all, or even by some aspirant for excellence not at all "to the manor born"—some untutored boy, whose later life was given to study and thought? That early training, early education, affords the possessor advantages cannot be denied; that preliminary qualification is almost essential for great enterprises, or high attainments in scientific or professional life, will be readily conceded, and much of what is said about medical education is not only true, but demands attention and action; so while we make no attack upon others' views, we still have a few words of our own to add.

There is much talk about attainment in students before being admitted to the practice of medicine, but who speaks about our attainments and means of culture when once fairly passed the examining board? The knowledge of a young man when he has conferred upon him the degree of Doctor in Medicine, is only supposed to be the mere rudiments of his profession; it is never anything greater. He has just learned how to learn, and obtains the legal right to use what he knows. If this fact can be impressed upon him, it will be worth more than any other view he can have of himself. In after life we obtain our medical education, and the physician who considers and acts upon the belief that he has completed it at graduation, is a quack all his life, though his diploma screen him from passing by his true name. Young men receive the credentials of some medical college, acknowledged to be only certificates of having studied medicine three years, and attended lectures as prescribed by law, and they pass into the practice of medicine, they are regarded as physicians, and receive the confidence of the community as such, almost regardless of other facts in relation to them. In this, the community betray their inability to judge, their ignorance of the essential grounds upon which confidence is based, and generally accept the fact of graduation as proof of qualification. This is perhaps all right in most instances, in all cases where the credentials have been fairly earned. Years of study, years of thought and reflection, of investigation and comparison, are the only means of perfecting in any degree our knowledge of disease and its best means of relief or cure; and he who graduates in medicine and ever after pursues his profession as a trade, estimating his suceess by the amount of his professional income, and devoting his energies to various and diverse objects, rather than to the study of his profession, is not a physician, and should no more be allowed to practice his art, than should the ignorant pretender, who, stationed by his side, compounds mixtures of indescribable virtues, or vends indiscernable morsels. It is no use for us to pretend we are physicians because we have received diplomas; calling a man Doctor, does not make him a physician—giving him a diploma, does not prevent him from being a quack.

Partial knowledge—knowledge simply of the proper dose and usual effects of a few medicinal substances, and of conditions of the system in which they may not generally do much harm, if administered, has long enough insured the possessor the honorable title of Doctor, without fear of contradiction; until so common and so easily obtained distinction comes at last to be almost a disgrace.

The real reason, then, that our standard in medical education is low, must be looked for not altogether in preliminary qualification and easy graduation, though this is powerful in degrading professional rank, but that other influence of which we speak, overshadows all other influences, and is *the* directing hand which has guided our professional course so nearly upon the level with empiricism and charlatanry. If there cannot be awakened in physicians generally a greater love of knowledge, and a firmer purpose to pursue it from a better estimate of the value of truth; if those who practice physic cannot learn to separate truth from error with discerning hand, and dare to speak it, then our standard must continue low; it must continue low until those who bear it, seek its elevation to the exclusion of everything else—commerce, trade, manufacturing, politics, pleasure, everything, and then the standard of medical education will not *remain* low, even though graduation is easy and medical colleges bestow their honors almost gratuitously.

It will not be inferred from our remarks that we would underrate the qualifications of the young physician; on the contrary we believe that often times graduation is the point of highest attainment ever reached, and that from it by a gradually descending scale he grows to know more of everything else and less of medicine—bestows attention to his profession sufficient only to imbibe and adopt error, and never scrutinizes his opinions, or considers the grounds upon which they are based; *experience* has great advantages, but it is liable to confirm error, as well as to establish truth.

Ever faithful janitors may guard the entrance to our profession, but beyond the doors are various highways opened up for the traveler; and facts warrant the belief, that within its barred gates, as without, ignorance, cupidity and quackery are found in almost uniform proportions. We have sometimes thought diplomas were screens for ignorance and incapacity, rather than indices of merit and worth, and that if wholly abandoned would leave the public to protect themselves by better discrimination, and physicians to earn their honors by industry and effort. At present they prove nothing, and those best acquainted with their value, accept them only for what they are worth; simply placing a man in the way of being a true physician and little else. We shall be happy to see discovered some plan of remedy for the evils which prevail in the practice of medicine, and if more stringent and impartial examinations will prove the specific, all hail! but if on the contrary it shall appear that our system of making physicians by giving certificates of study, is all a farce and delusion, and that those only are true physicians who devote their attention, thought and effort to obtaining medical knowledge, then also, all hail! for the whole world of medicine requires regeneration. It is not proposed, however, to change the plan of instruction, to find any fault with its general operation, or to indicate any other system or standard; it is only to give utterance to some general and pretty well understood facts, hoping that good may grow out of a frank expression of truths which in themselves are quite familiar to all careful observers.

